# Transcranial sonothrombolysis using high-intensity focused ultrasound: impact of increasing output power on clot fragmentation

**DOI:** 10.1186/2050-5736-1-22

**Published:** 2013-11-01

**Authors:** Golnaz Ahadi, Christian S Welch, Michele J Grimm, David J Fisher, Eyal Zadicario, Karin Ernström, Arne H Voie, Thilo Hölscher

**Affiliations:** 1 Brain Ultrasound Research Laboratory (BURL), University of California, San Diego, 200 West Arbor Drive, San Diego, CA 92103-8756, USA; 2 Department of Radiology, University of California, San Diego, 200 West Arbor Drive, San Diego, CA 92103-8756, USA; 3 Department of Family and Preventive Medicine, University of California, San Diego, 200 West Arbor Drive, San Diego, CA 92103-8756, USA; 4 Department of Neurosciences, University of California, San Diego, 200 West Arbor Drive, San Diego, CA 92103-8756, USA; 5 Department of Biomedical Engineering, Wayne State University, Detroit, MI 48202, USA; 6 Neuro Program, InSightec, Inc., Tirat Carmel, Israel

**Keywords:** Thrombolysis, High-intensity focused ultrasound, Stroke, Clot fragmentation, Clot debris

## Abstract

**Background:**

The primary goal of this study was to investigate the relationship between increasing output power levels and clot fragmentation during high-intensity focused ultrasound (HIFU)-induced thrombolysis.

**Methods:**

A HIFU headsystem, designed for brain applications in humans, was used for this project. A human calvarium was mounted inside the water-filled hemispheric transducer. Artificial thrombi were placed inside the skull and located at the natural focus point of the transducer. Clots were exposed to a range of acoustic output power levels from 0 to 400 W. The other HIFU operating parameters remained constant. To assess clot fragmentation, three filters of different mesh pore sizes were used. To assess sonothrombolysis efficacy, the clot weight loss was measured.

**Results:**

No evidence of increasing clot fragmentation was found with increasing acoustic intensities in the majority of the study groups of less than 400 W. Increasing clot lysis could be observed with increasing acoustic output powers.

**Conclusion:**

Transcranial sonothrombolysis could be achieved *in vitro* within seconds in the absence of tPA and without producing relevant clot fragmentation, using acoustic output powers of <400 W.

## Background

The majority of strokes are ischemic, caused by intracranial thrombo-embolic arterial occlusion. Vessel recanalization is the primary goal of all acute stroke treatment approaches. Achieving vessel recanalization without causing further damage is a key objective in effective treatment. Innovative recanalization strategies or options to improve tPA efficacy are of high interest. Mechanical (i.e., mechanical embolism removal cerebral ischemia, MERCI) and chemical (i.e., tPA) methods to achieve successful thrombolysis have been evaluated with regard to efficacy and safety. With mechanical removal of a thrombotic occlusion, an undesirable side effect has been the potential harmful effects caused by clot fragments [1]. Clot fragments may lead to secondary vessel occlusion further downstream in the supply area of the affected vessel. This is a safety concern because of the potential risk of secondary strokes [2].

Basic principles using ultrasound (US) to enhance thrombolysis have been described [3-8], and first clinical studies on transcranial sonothrombolysis in stroke patients using diagnostic US devices are promising [9-12]. Current research in thrombolysis has been focused mainly on clot lysis feasibility [13-17], but only to a limited extent on clot fragmentation or other potential side effects, such as unwanted temperature elevation in the tissue. To date, only a few publications are available describing the impact of mechanical versus pharmacological recanalization strategies on clot fragmentation [18], the concomitant effects of potential heating [19], or the specific effects of focused ultrasound in this regard [20,21].

The goal of this study was to investigate the impact of increasing acoustic output powers on potential clot fragmentation, using a novel transcranial high-intensity focused ultrasound (HIFU) headsystem.

## Methods

### Description of the HIFU headsystem

For all studies, a HIFU headsystem (ExAblate™ 4000, InSightec, Inc., Tirat Carmel, Israel) equipped with a hemispheric transducer was used. Due to its design, the multi-element array produces a sharp focus in the geometrical center of the hemispheric transducer, which can be steered electronically as well as mechanically. A detailed description of the device is available in a contribution recently published by the same group [22].

### Experimental setup

**Figure 1:**
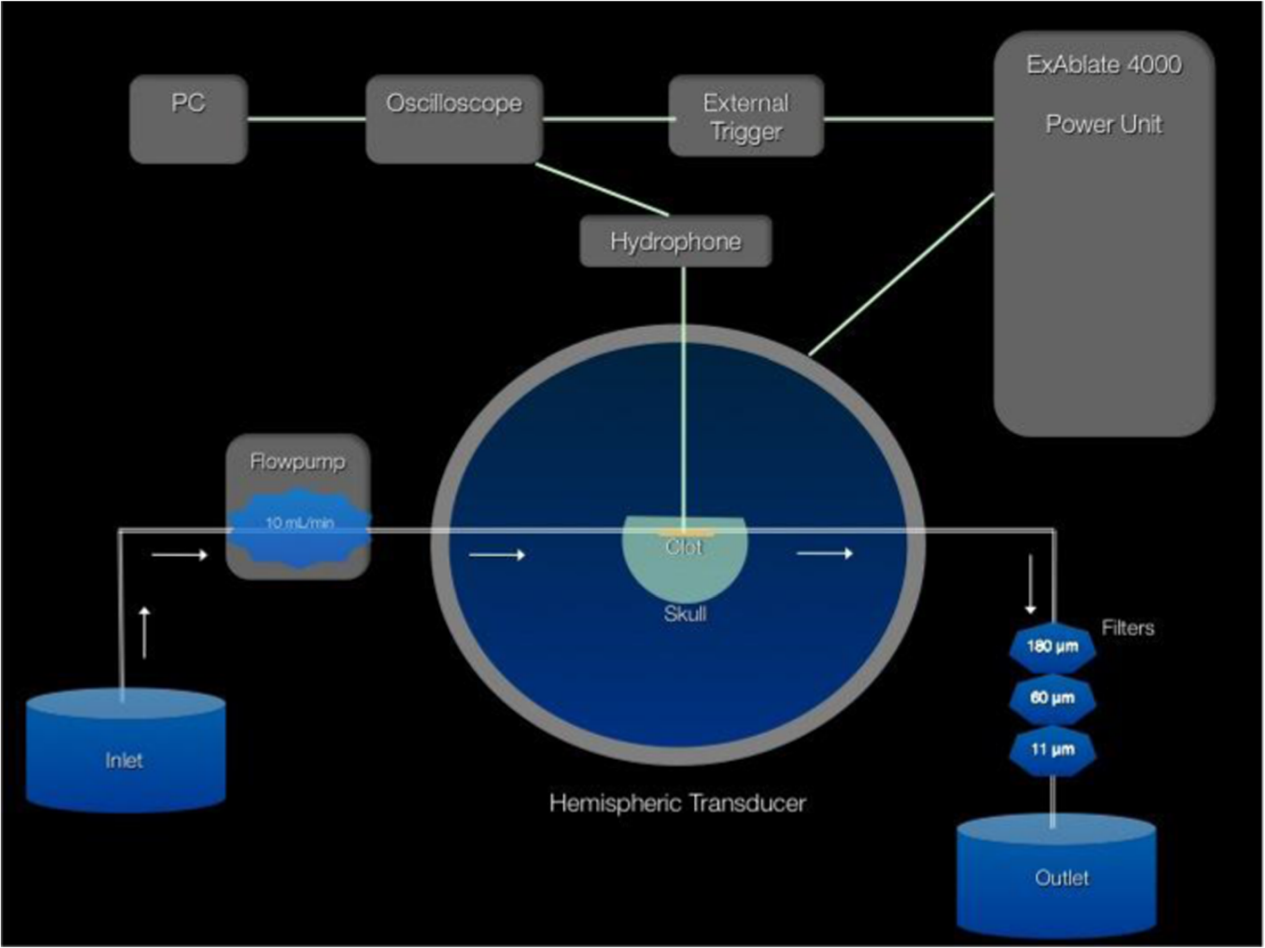
**Schematic display of the *in vitro *HIFU thrombolysis and clot fragmentation experimental setup.** The hydrophone position describes the location of the natural focus beam, in the center of the blood clot.

A human cadaveric skull was degassed for 72 h prior to being mounted upside down to the bottom of an acrylic plate, which covered the hemispheric transducer. The plate had a hole in its center (∅16 cm). The hemispheric transducer was filled with molecular grade (distilled and deionized) water that was degassed for a period of 2 h immediately prior to use. A human cadaveric skull was provided by the University of California, San Diego (UCSD) Division of Anatomy. Venous whole blood was drawn from healthy unmedicated donors, per UCSD-approved IRB protocol, into vacutainer citrate tubes. Clots were generated around a silk thread after adding CaCl_2_ and incubated for 3.0 h at 37°C. The average clot weight was 0.2652 g ± 6%. A detailed description of the clot preparation has been published by the same group recently [22]. The experimental setup is displayed in Figure 1.

### Ultrasound parameter settings

A multi-location insonation pattern was used in all experiments. First, the thrombus was positioned in such a way that its center was aligned with the natural focus of the HIFU system (X/Y/Z, 0/0/150 mm). For all sonothrombolysis experiments, the HIFU focus was then electronically steered along the longitudinal axis of the thrombus, aiming at nine different locations with a stepwidth of 2.0 mm to cover the entire length of the thrombus (start at −10/0/150 mm; stop at +10/0/150 mm). The insonation duration for all experiments, regardless of acoustic output power, was limited to 30 s.

The ultrasound parameters were chosen for efficacious clot lysis obtained based on previous first data on *in vitro* HIFU sonothrombolysis using the ExAblate™ 4000 headsystem [22]. Accordingly, the duty cycle and pulse length were kept constant at 50% and 200 ms, respectively, for all data collection points. The only varying factor of the HIFU setup was the change of the acoustic power, ranging from 0 to 400 W (0, 50, 100, 125, 150, 200, 235, 270, and 400 W). The varying acoustic powers were used to evaluate the relation between insonation intensities and effect on clot lysis and fragmentation.

### Assessment of clot lysis efficacy and fragmentation

After the 30 s of insonation, the flow was continued for an additional 2 min, and the solution was collected in a beaker. The thrombi were weighed pre- and post-insonation. After incubation, the pre-weight was assessed by placing the clot, attached to the string, on a precision scale (model ML303E, Mettler Toledo, Inc., Greifensee, Switzerland). After insonation the post-weight was assessed by pulling the string to remove the attached thrombus out of the test tube. The clot including the string was then placed on the scale and weighed again. Subtracting the post-weight from the pre-weight, the percentage weight loss for each individual clot and the average percentage weight loss for each study group were calculated and recorded. Before placing the solution in the beaker, it passed three differently sized mesh filters (Millipore, Tullagreen Carrigtwohill, County Cork, Ireland) with mesh widths of 180, 60, and 11 μm to capture the clot fragments. The serial filtration with descending mesh size filters was set up to represent small arteries as well as the microvasculature. The microvasculature of the human brain varies in its cross-sectional diameter, usually with a diameter of <10 μm, which is reported to be subcapillary [23-25]. The reason why 11-μm mesh size was chosen in the present study was mainly due to accessibility and the pore size being close enough to emulate the size of the vessels in the capillary range. The amount of clot fragmentation per filter size was calculated by subtracting the pre-wet filter weights from the post-wet filter weights with the difference documented in percent clot weight. To do so, the filters were soaked in degassed deionized water for 2.5 min prior to the experiment. To remove the entrapped water drops, the filter's edge was tapped twice on gauze. After tapping, the filter's weight was assessed and defined as the *pre*-wet filter weight. Following the ultrasound exposure, the filters were tapped again in the same manner and the *post*-wet filter weight was assessed. If the sum of the post-wet filter weight *minus* the pre-wet filter weight was greater than ‘0’,, an assumption was made that the clot fragmentation occurred.

### Acoustic measurements

Without the test tubing in place, for all experimental groups, the acoustic parameters spatial peak, temporal average intensity (*I*_SPTA_), peak negative pressure (*P*_neg_), and peak positive pressure (*P*_pos_) were measured first using a HIFU hydrophone (model Y120, Sonic Concepts, Seattle, WA, USA), calibrated for the frequency of 220 kHz. To account for the interference of the tubing itself, the acoustic measurements were repeated by placing the hydrophone at focus inside the tubing.

### Statistical analysis

For efficacy, the aim was to establish if weight loss (in percent and gram) was different among groups. A linear regression model was used to examine if there is a difference in the mean weight loss among groups (primarily, is each group is different from the 0 W group).

For fragmentation, the aim was to establish if the clot fragmentation (post-/pre-filter weight) was different among groups for each separate filter size (11, 60, and 180 μm). Wilcoxon rank-sum tests were used to examine if the clot fragmentation in each group (50, 100, 150, 200, 235, 270, and 400 W) is different from the clot fragmentation in the 0 W group. The *p* values were adjusted using the Holm's procedure to correct for multiple comparisons. Descriptive statistics and boxplots for overall and group clot fragmentation were provided.

## Results

### Clot fragmentation

A total of *N* = 352 clots were studied, divided into nine subgroups of increasing acoustic output powers. To test for clot fragmentation, three different filter sizes were used. For the 400 W as well as for the 150-W acoustic output power group, a statistical significant clot fragmentation could be observed for the 180-μm filter size. For 60 and 11 μm groups, as well as for any other study group or filter size, no statistical significant clot fragmentation was observed when compared to the control group (0 W). Detailed statistical findings of all intensity groups and pore sizes are given in Tables 1, 2, and 3, respectively.

**Table 1:** Clot fragmentation (post-/pre-wet filter weight) 180-μm filter

**Group**	**Acoustic output power (W)**	**Number**	**Mean weight (mg)**	**Standard deviation**	**Min (mg)**	**Median (mg)**	**Max (mg)**	***P *** **Value**
1	0	60	−4.2	0.01	−18.5	−5.5	8.5	-
2	50	62	−2.6	0.01	−13.5	−3	15.5	0.6666
3	100	20	−1	0.01	−10.5	0	7.5	0.1285
4	125	63	−1.7	0.01	−15.5	−1.5	14.5	0.1134
5	150	65	−1.2	0.01	−12.5	−1.5	10.5	0.0266
6	200	20	18.3	0.1	−17.5	−3	433.5	0.6666
7	235	20	−2.8	0.01	−10.5	−3.5	6.5	0.6666
8	270	22	−1.3	0.01	−12.5	1.5	15.5	0.2868
9	400	20	2	0.01	−17.5	0	18.5	0.0048
Overall	-	352	0	0.02	20	0	0.43	-

*p* Values reflect comparison to the 0 W group, adjusted for multiple comparison.

**Table 2:** Clot fragmentation (post-/pre-wet filter weight) 60-μm filter

**Group**	**Acoustic output power (W)**	**Number**	**Mean weight (mg)**	**Standard deviation**	**min (mg)**	**Median (mg)**	**Max (mg)**	***p *** **Value**
1	0	60	3.7	0	−1.1	2.9	21.9	-
2	50	62	3.5	0	−1.1	2.4	10.9	>0.9999
3	100	20	1.3	0.04	1.9	3.9	18.1	>0.9999
4	125	63	3.3	0	−4.1	2.9	11.9	>0.9999
5	150	65	3.2	0	−12.1	2.9	15.9	>0.9999
6	200	20	21.2	0.06	−2.1	3.4	19.1	>0.9999
7	235	20	2.6	0	−2.1	1.9	6.9	>0.9999
8	270	22	10.6	0.04	−6.1	3.4	180.9	>0.9999
9	400	20	13.4	0.04	−1.1	3.9	190.9	>0.9999
Overall	-	352	0.01	0.02	−10	0	0.19	-

*p* Values reflect comparison to the 0 W group, adjusted for multiple comparison.

**Table 3:** Clot fragmentation (post-/pre-wet filter weight) 11-μm filter

**Group**	**Acoustic output power (W)**	**Number**	**Mean weight (mg)**	**Standard deviation**	**Min (mg)**	**Median (mg)**	**Max (mg)**	***P *** **Value**
1	0	60	2.4	0	−8.2	1.8	11.8	-
2	50	62	3.3	0	−8.2	2.8	19.8	>0.9999
3	100	20	18.5	0.07	−2.2	3.3	310.8	>0.9999
4	125	63	2.7	0	−6.2	2.8	13.8	>0.9999
5	150	65	4.2	0.01	−6.2	2.8	50.8	>0.9999
6	200	20	3	0	−3.2	2.8	12.8	>0.9999
7	235	20	2.2	0	−2.2	1.8	5.8	>0.9999
8	270	22	2.2	0	−3.2	2.3	9.8	>0.9999
9	400	20	2.7	0	−1.2	1.8	9.8	>0.9999
Overall	-	352	3.9	0.02	−8.2	2.8	310.8	-

*p* Values reflect comparison to the 0 W group, adjusted for multiple comparison.

### Sonothrombolysis efficacy

**Figure 2:**
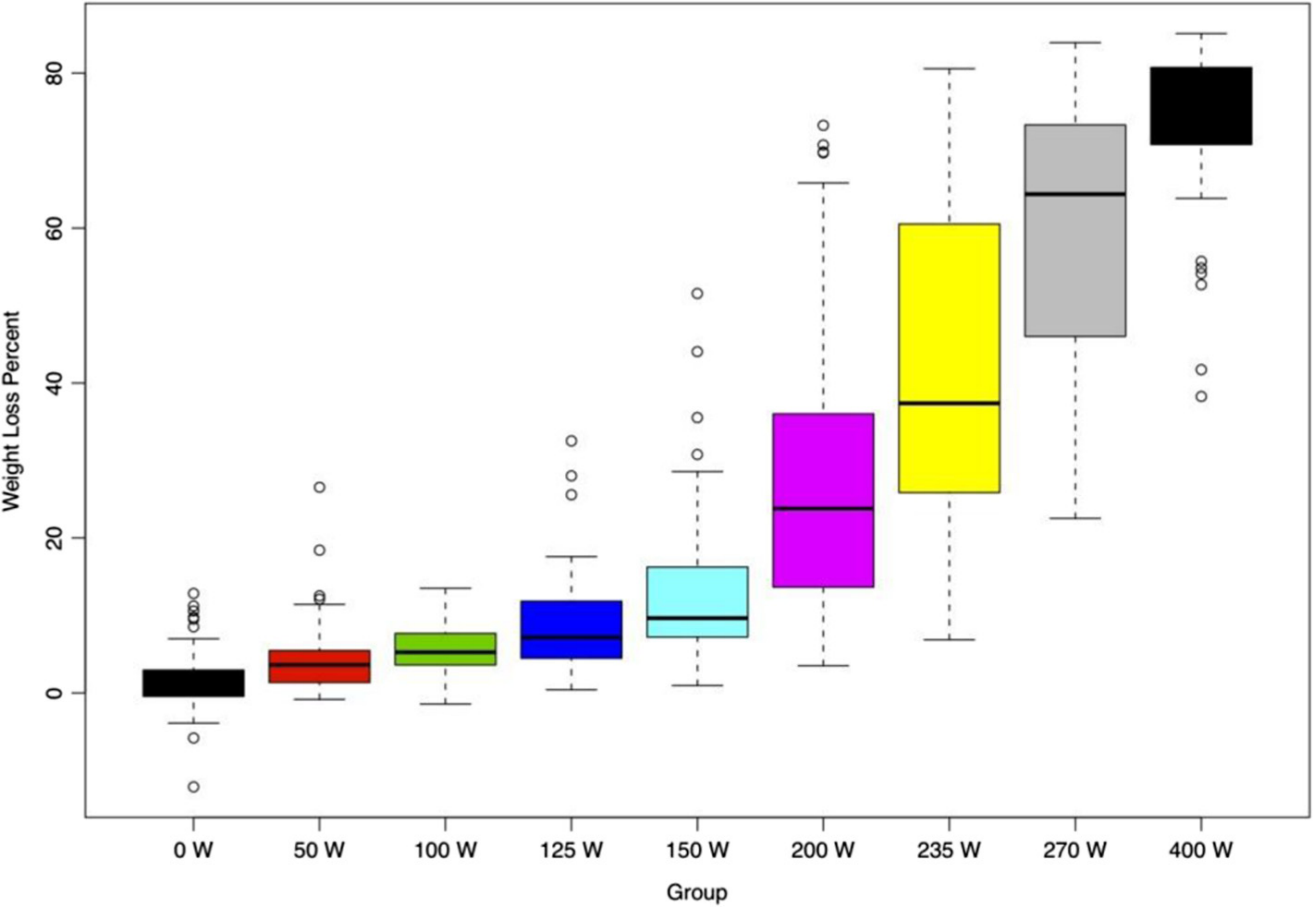
Percent clot weight loss for each acoustic output power group.

A total of *N* = 561 clots were studied. For groups 4 to 9, a statistically significant (*p* < 0.001) weight loss could be achieved (Table 4). For groups 1 to 3, no significant (*p* > 0.05) weight loss could be seen. A visual presentation of the efficacy results is given in Figure 2.

**Table 4:** Percent clot weight loss - clot lysis in relation to intensity

**Group**	**Acoustic output power (W)**	**Number**	**Mean weight loss (mg)**	**Standard deviation**	**Mean weight loss (%)**	**Standard deviation**	***P *** **Value**
1	0	60	0.0	0.01	1.75	4.01	>0.05
2	50	62	10.0	0.01	4.55	4.62	>0.05
3	100	66	10.0	0.01	5.63	3.22	>0.05
4	125	63	20.0	0.02	8.97	6.3	<0.001
5	150	65	30.0	0.02	12.9	9.75	<0.001
6	200	61	70.0	0.05	28.22	18.70	<0.001
7	235	61	100.0	0.05	41.41	20.3	<0.001
8	270	62	150.0	0.04	61.04	14.56	<0.001
9	400	61	180.0	0.03	74.83	10.12	<0.001

### Ultrasound parameters/acoustic properties

For each acoustic output power value, the *I*_spta_, the peak negative pressure (*P*_neg_), the peak positive pressure (*P*_pos_), and the total energy in kilojoules were measured at focus with the tubing in place. In comparison to the acoustic measurements without the tubing in place, we measured the *I*_spta_ and subsequently the energy to be 89.8% while the *P*_neg_ and *P*_pos_ values were 93.1%. Table 5 provides an overview of the acoustic data with the test tubing in place.

**Table 5:** Acoustic parameters at the focus with tubing

**Ac power (W)**	***I***_**spta **_**(W/cm**^**2**^**)**	***P***_**neg **_**(MPa)**	***P***_**pos **_**(MPa)**	**Energy (kJ)**
0	0.00	0.00	0.00	0.00
50	29.71	1.45	1.32	0.14
100	59.27	2.07	1.93	0.28
125	75.57	2.34	2.11	0.35
150	92.05	2.55	2.34	0.42
200	121.05	2.95	2.73	0.57
235	144.22	3.27	2.91	0.66
270	130.43	3.81	3.09	0.76
400	193.24	4.32	3.76	1.13

## Discussion

Clot fragmentation during thrombolytic therapy using ultrasound is a safety concern. It could be demonstrated in the present study that sonothrombolysis using HIFU can be achieved with great efficiency without causing significant clot fragmentation using acoustic output powers of less than 400 W.

An undesirable side effect of sonothrombolysis has been the potential harmful effects caused by clot fragments, which may lead to secondary vessel occlusion. If the fragments produced from successful US-induced recanalization are large in size, they may limit the blood flow farther downstream, causing secondary embolic strokes. To date, the knowledge about sonothrombolysis strategies and resulting clot fragmentation is sparse. The goals of the present work were to collect data on clot fragments that were produced subsequent to thrombolysis using HIFU and in absence of tPA and to quantitatively evaluate the resultant fragment size distribution. Current literature addresses clot fragmentation as it relates to vessel size. During their *in vitro* sonothrombolysis experiments, Rosenschein et al. [20] examined clot fragmentation by performing post-insonation, a unidirectional saline flush through a segment of an *ex vivo* bovine artery and three differently pore-sized filters. The group could demonstrate that irrelevant of the *I*_spta_ delivered by their HIFU system, 93% of the fragment material was subcapillary. In this study, subcapillary was defined to be a material smaller than 8 μm in size.

A similar serial filtration setup was used in the present *in vitro* study to examine clot fragmentation as a result of HIFU-induced sonothrombolysis. Using three differently sized mesh filters (180, 60, and 11 μm), pre-/post-filter weights were assessed. Given the slightly different filter sizes used in the present study, relevant clot fragmentation was found at elevated acoustic energies and for the large filter size only, confirming to a great extent the findings of Rosenschein et al. For the 180-μm filter size, a statistically significant *p* value (>0.05) was found for 150- and the 400-W experimental groups, suggesting that clot fragmentation was significantly greater than the control for this specific filter size. For the 11- and 60-μm-sized filters, no statistically significant clot fragmentation occurred in the experimental groups when compared to the control groups. For the 150 W group, the median value for the 180-μm filter size was negative (post-wet filter weight − pre-wet filter weight). Negative median values would imply that the filter weight prior to the experiment would have been higher than the post-filter weight. Since the filters cannot lose weight during the experiment, the most reasonable explanation for this might have been the relative inaccuracy of the procedure itself. Prior to the experiment, each filter was soaked in water. In preparation for the actual experiment, the edge of the filters was tapped twice on gauze using blunt forceps to remove larger drops of water. Post insonation, the filters were tapped again in the same manner. This appeared to be the most reasonable procedure to equalize the conditions of filter weight assessment pre-/post-insonation. The procedure itself, however, bares a limited sensitivity. This might explain why ‘negative’ filter weights were seen. Due to this, and although formally statistically significant, the findings in the 150 W group were interpreted as most likely to be not of potential clinical relevance. Aside from this, the median filter weight for the 400 W group was found to be significantly greater for the 180-μm filter size in comparison to the median filter weight of either the control or any other group. Therefore, this finding was found to be significant and was most likely clinically relevant. The fact that clot fragmentation was seen only in the highest intensity group and with the largest filter size suggests that larger pieces of the clot were torn apart most likely due to the visible and vigorous displacement of the clot inside the test tube during insonation. Similar observations regarding pulsed focused ultrasound-induced displacements of *in vitro* blood clots were recently described by Wright et al. [26]. For the two smaller mesh filter sizes, no statistically significant differences in pre-/post-filter weights could be seen, independent from the acoustic output power or thrombolytic efficacy. This finding suggests that if clot fragmentation might have occurred, the fragments were smaller than 11 μm. A possible reason for the small amount of detectable clot fragmentation with regard to HIFU thrombolysis was given by Maxwell et al. [27]. The group suggested the creation of cavitation clouds at focus in which the clot fragments might be entrapped and further fractionated even in the presence of directional flow.

Despite the promising findings of the present work with regard to clot fragmentation and clot lysis, the data has to be interpreted with great care. Whether or not the potential of using HIFU for transcranial sonothrombolysis without causing adverse side effects due to clot fragmentation would suggest a rather safe vessel recanalizing method has to be verified in appropriate animal models first before it might be considered for human application. From current sonothrombolysis trials, we have learned that mainly secondary hemorrhage is a safety concern and might diminish the potential of early recanalization using transcranial ultrasound. Secondary hemorrhage has been described mainly in combination with low-frequency ultrasound and to lesser extent with diagnostic range frequencies of 1.0 MHz or above [9,10,12,28]. It should be mentioned that the majority of the present sonothrombolysis trials have been performed with diagnostic range ultrasound devices and in combination with tPA. Clinical trial data using focused ultrasound devices and in absence of tPA does not exist.

### Limitations of the present study and future outlook

The experimental setup represents an *in vitro* sonothrombolysis model of efficacy assessment and resultant clot fragmentation. Thus, future sonothrombolysis efficacy studies will have to be performed in an appropriate *in vivo model,* providing quantitative analysis of potential clot fragmentation postmortem. The blood clots used in this study were artificially made using blood from healthy human volunteers. Accordingly, these blood clots are a limiting element for adequate demonstration of results for thrombolysis efficiency and clot fragmentation. Future experiments should incorporate the use of *ex vivo* thrombi taken from sufferers of an occluded vessel (harvested during neurointerventional procedures in actual stroke patients) [2]. Furthermore, besides fragmentation, cavitation and thermal effects have to be studied in depth due to the safety concerns. It has been shown that cavitation may lead to microvessel disruption, causing potential intracranial hemorrhages [29]. Thermal effects are of concern with regard to heat-related tissue damage. Future safety experiments will have to focus on the effects of stable and inertial cavitation both inside and outside the vessel as well as thermal effects of transcranial sonothrombolysis.

The ultimate goal is to move the *in vitro* and animal model studies to the clinical application in humans. In order to do so, the HIFU system will have to be combined with a magnetic resonance imaging (MRI) system for neuronavigation. Since the HIFU brain system does not provide imaging capabilities, it is - in the clinical setup - an integrative part of an MRI scanner to navigate the focus beam towards the target structure. The time to prepare the patient and the high cost to use these two devices on stroke sufferers might be the current limiting factors for therapeutic clinical use of this technology on a broader scale, except in very specialized comprehensive stroke centers. However, the potential impact of MRI-guided HIFU in clinical use for the treatment of ischemic stroke in the absence of therapeutic lytic agents is significant. Not using lytic agents in combination with US will result in avoidance of the side effects of these therapeutics, such as tPA-induced hemorrhages. Of great importance as well is the fact that a much larger stroke population who are not eligible for tPA therapy might benefit from sonothrombolytic treatment using transcranial HIFU.

## Conclusion

Using a first clinical transcranial HIFU headsystem, it has been demonstrated *in vitro* that transcranial sonothrombolysis using HIFU can be achieved within seconds in the absence of tPA and without significant clot fragmentation, except for high acoustic output powers beyond 400 W. Future research in this field would have to demonstrate the translation of this potential new therapeutic approach and the reproducibility of transcranial HIFU sonothrombolysis *in vivo*. More importantly, the safety of HIFU has to be shown with the optimized parameters in an appropriate *in vivo* model.

## Competing interests

The authors declare that they have no competing interests.

## Authors’ contributions

GA designed the experimental setup, acquired all data, and drafted the manuscript. CW and GA acquired the data and drafted/edited the manuscript. MG revised the manuscript and contributed editorially. DF acquired data with CW and GA, supervised GA/CW in all experimental aspects, and revised the manuscript. KE did the statistical analysis. EZ provided the technical support for the HIFU system. AV is involved in the experimental planning and performed all acoustic measurements. TH initiated the study, provided the concept and design, supervised/monitored all parts of the project, and revised/re-edited the manuscript. All authors read and approved the final manuscript.

## References

[B1] Dumont TM Mokin M Sorkin GC Levy EI Siddiqui AH 2013 Journal of Neurointerventional Surgery: Aspiration thrombectomy in concert with stent thrombectomy doi:10.1136/neurintsurg-2012-010624.rep. 10.1136/neurintsurg-2012-010624.rep 23868216

[B2] Marder VJ Chute DJ Starkman S Abolian AM Kidwell C Liebeskind D Ovbiagele B Vinuela F Duckwiler G Jahan R Vespa PM Selco S Rajajee V Kim D Sanossian N Saver JL Analysis of thrombi retrieved from cerebral arteries of patients with acute ischemic stroke Stroke 2006 37 8 2086 93 doi:10.1161/01.STR.0000230307.03438.94. 10.1161/01.STR.0000230307.03438.94 16794209

[B3] Prokop AF Soltani A Roy RA Cavitational mechanisms in ultrasound-accelerated fibrinolysis Ultrasound Med Biol 2007 33 6 924 33 10.1016/j.ultrasmedbio.2006.11.022 17434661

[B4] Braaten JV Goss RA Francis CW Ultrasound reversibly disaggregates fibrin fibers Thromb Haemost 1997 78 3 1063 68 9308755

[B5] Devcic-Kuhar B Pfaffenberger S Gherardini L Mayer C Groschl M Kaun C Benes E Tschachler E Huber K Maurer G Wojta J Gottsauner-Wolf M Ultrasound affects distribution of plasminogen and tissue-type plasminogen activator in whole blood clots *in vitro* Thromb Haemost 2004 92 5 980 85 15543323 10.1160/TH04-02-0119

[B6] Datta S Ammi AY Coussios CC Holland CK Monitoring and simulating stable cavitation during ultrasound-enhanced thrombolysis J Acoust Soc Am 2007 122 3052 3052 52

[B7] Datta S Coussios CC McAdory LE Tan J Porter T De Courten-Myers G Holland CK Correlation of cavitation with ultrasound enhancement of thrombolysis Ultrasound Med Biol 2006 32 8 1257 67 doi:10.1016/j.ultrasmedbio.2006.04.008. 10.1016/j.ultrasmedbio.2006.04.008 16875959 PMC1937506

[B8] Hitchcock KE Ivancevich NM Haworth KJ Caudell Stamper DN Vela DC Sutton JT Pyne-Geithman GJ Holland CK Ultrasound-enhanced rt-PA thrombolysis in an *ex vivo* porcine carotid artery model Ultrasound Med Biol 2011 37 8 1240 51 10.1016/j.ultrasmedbio.2011.05.011 21723448 PMC4025997

[B9] Alexandrov AV Mikulik R Ribo M Sharma VK Lao AY Tsivgoulis G Sugg RM Barreto A Sierzenski P Malkoff MD Grotta JC A pilot randomized clinical safety study of sonothrombolysis augmentation with ultrasound-activated perflutren-lipid microspheres for acute ischemic stroke Stroke 2008 39 5 1464 69 10.1161/STROKEAHA.107.505727 18356546 PMC2707058

[B10] Alexandrov AV Molina CA Grotta JC Garami Z Ford SR Alvarez-Sabin J Montaner J Saqqur M Demchuk AM Moyé LA Hill MD Wojner AW CLOTBUST Investigators Ultrasound-enhanced systemic thrombolysis for acute ischemic stroke N Engl J Med 2004 351 21 2170 78 10.1056/NEJMoa041175 15548777

[B11] Eggers J Koch B Meyer K Konig I Seidel G Effect of ultrasound on thrombolysis of middle cerebral artery occlusion Ann Neurol 2003 53 6 797 800 10.1002/ana.10590 12783427

[B12] Eggers J Konig IR Koch B Handler G Seidel G Sonothrombolysis with transcranial color-coded sonography and recombinant tissue-type plasminogen activator in acute middle cerebral artery main stem occlusion: results from a randomized study Stroke 2008 39 5 1470 75 10.1161/STROKEAHA.107.503870 18340100

[B13] Alonso A Dempfle CE Della Martina A Stroick M Fatar M Zohsel K Allémann E Hennerici MG Meairs S *In vivo* clot lysis of human thrombus with intravenous abciximab immunobubbles and ultrasound Thromb Res 2009 124 1 70 4 10.1016/j.thromres.2008.11.019 19349068

[B14] Meunier JM Holland CK Lindsell CJ Shaw GJ Duty cycle dependence of ultrasound enhanced thrombolysis in a human clot model Ultrasound Med Biol 2007 33 4 576 83 10.1016/j.ultrasmedbio.2006.10.010 17337113 PMC1995459

[B15] Meairs S Culp W Microbubbles for thrombolysis of acute ischemic stroke Cerebrovasc Dis 2009 27 Suppl 2 55 65 19372661 10.1159/000203127

[B16] Porter TR LeVeen RF Fox R Kricsfeld A Xie F Thrombolytic enhancement with perfluorocarbon-exposed sonicated dextrose albumin microbubbles Am Heart J 1996 132 5 964 68 10.1016/S0002-8703(96)90006-X 8892768

[B17] Pieters M Hekkenberg RT Barrett-Bergshoeff M Rijken DC The effect of 40 kHz ultrasound on tissue plasminogen activator-induced clot lysis in three *in vitro* models Ultrasound Med Biol 2004 30 11 1545 52 10.1016/j.ultrasmedbio.2004.08.028 15588966

[B18] Greenberg RK Ouriel K Srivastava S Shortell C Ivancev K Waldman D Illig K Green R Mechanical versus chemical thrombolysis: an *in vitro* differentiation of thrombolytic mechanisms J Vasc Interv Radiol 2000 11 2 Pt 1 199 205 10716390 10.1016/s1051-0443(07)61465-1

[B19] Hartnell GG Saxton JM Friedl SE Abela GS Rosenschein U Ultrasonic thrombus ablation: *in vitro* assessment of a novel device for intracoronary use J Interv Cardiol 1993 6 1 69 76 10.1111/j.1540-8183.1993.tb00443.x 10150988

[B20] Rosenschein U Furman V Kerner E Fabian I Bernheim J Eshel Y Ultrasound imaging-guided noninvasive ultrasound thrombolysis: preclinical results Circulation 2000 102 2 238 45 10.1161/01.CIR.102.2.238 10889137

[B21] Wright C Hynynen K Goertz D 2012 Invest Radiol: In vitro and in vivo high-intensity focused ultrasound thrombolysis doi:10.1097/RLI.0b013e31823cc75c. 10.1097/RLI.0b013e31823cc75c PMC3302946 22373533

[B22] Holscher T Fisher D Raman R Ernstrom K Zadicario E Bradley W Voie A 2011 Journal of Neurology and Neurophysiology: Noninvasive transcranial clot lysis using high intensity focused ultrasound doi:10.4172/2155-9562.S1-002.

[B23] Moody DM Brown WR Challa VR Stump DA Reboussin DM Legault C Brain microemboli associated with cardiopulmonary bypass: a histologic and magnetic resonance imaging study Ann Thorac Surg 1995 59 5 1304 07 10.1016/0003-4975(95)00057-R 7733757

[B24] Hudetz AG Blood flow in the cerebral capillary network: a review emphasizing observations with intravital microscopy Microcirculation 1997 4 2 233 52 10.3109/10739689709146787 9219216

[B25] Kulik T Kusano Y Aronhime S Sandler AL Winn HR Regulation of cerebral vasculature in normal and ischemic brain Neuropharmacology 2008 55 3 281 88 10.1016/j.neuropharm.2008.04.017 18541276 PMC2896303

[B26] Wright CC Hynynen K Goertz DE Pulsed focused ultrasound-induced displacements in confined *in vitro* blood clots IEEE Trans Biomed Eng 2012 59 3 842 51 doi:10.1109/TBME.2011.2180904. 22194235 10.1109/TBME.2011.2180904 PMC4677835

[B27] Maxwell AD Cain CA Duryea AP Yuan L Gurm HS Xu Z Noninvasive thrombolysis using pulsed ultrasound cavitation therapy - histotripsy Ultrasound Med Biol 2009 35 12 1982 94 10.1016/j.ultrasmedbio.2009.07.001 19854563 PMC2796469

[B28] Daffertshofer M Gass A Ringleb P Sitzer M Sliwka U Els T Sedlaczek O Koroshetz WJ Hennerici MG Transcranial low-frequency ultrasound-mediated thrombolysis in brain ischemia: increased risk of hemorrhage with combined ultrasound and tissue plasminogen activator: results of a phase II clinical trial Stroke 2005 36 7 1441 46 10.1161/01.STR.0000170707.86793.1a 15947262

[B29] Kennedy JE Ter Haar GR Cranston D High intensity focused ultrasound: surgery of the future? Br J Radiol 2003 76 909 590 99 10.1259/bjr/17150274 14500272

